# Reasons behind stymied public hospital governance reform in China

**DOI:** 10.1371/journal.pone.0222204

**Published:** 2019-09-09

**Authors:** Sheng Nong, Nengliang Aaron Yao

**Affiliations:** 1 You Jiang Medical University for Nationalities, Baise, China; 2 Peking University, School of Public Health, Beijing, China; 3 School of Health Care Management (Key Lab of Health Economics and Policy, National Health Commission), Jinan, China; 4 University of Virginia, Department of Public Health Sciences, Charlottesville, Virginia, United States of America; University Medical Center of Princeton at Plainsboro, UNITED STATES

## Abstract

**Background:**

The public hospital governance reform in China is pledged to improve the governance of public hospitals and deliver affordable and high-quality care. However, progress in public hospital reform has been slow. The reason is poorly understood.

**Methods:**

A research center affiliated with China National Health Commission has conducted 32 workshops to interview 124 public hospital administrators from 30 provincial-level administrative divisions and 105 various-level government officials from three provinces. About 80% of administrators and 78% officials actively participated the discussions. We used a descriptive theoretical approach to understand the relationships between the governance reform and characteristics of its stakeholders. We also analyzed stakeholder interests and their power to influence the reform.

**Findings:**

About 66% of hospital administrators, 72% of health officials, and less than 10% of other officials support a new hospital governing structure. Local leadership, hospital administrators, and health commission said that administrators should have more power over the management of public hospitals. Other government departments and healthcare professionals had reservations on the governance reform. The reform of public hospital governance faces significant obstacles. The interests of most government stakeholders are not aligned with public interests. All stakeholders perceived that their workload would increase in the short term because of the governance reform of public hospitals. Most people involved in the reform are not incentivized to collaborate. The health commission has limited financial resources and insufficient political power to implement a massive reform. Most importantly, the public hospital reform is not, and likely will not be, a top policy priority to the central government or local leaderships.

**Interpretation:**

The health commission needs more political support and resources to speed up the public hospital reform. To fulfill the pledge of affordable, equitable access to quality care, Chinese government needs to overcome significant obstacles in the public hospital reform.

## Introduction

The recent healthcare reform in China aims to expand health insurance coverage, equalize public health services, strengthen primary care, establish essential medicines program, and reform public hospitals [1]. Most of the researchers agree that the social health insurance system is the most successful policy pillar in the reform [1–3]. Recently, a new State Medical Insurance Administration has been established to consolidate health insurance schemes [3]. In contrast to the success of the health insurance expansion, progress in healthcare delivery reform has been slow, especially with the public hospital-centered system [1].

The State Council issued the newest round of policies to improve public hospital governance structure in 2015. The reform emphasizes separation of government control and hospital management [1,2,4,5]. There are many challenges in implementing the separation of ownership from operation. In this study, the project team from a research center of China National Health Commission met with public hospital administrators from 30 provincial-level administrative divisions and various stakeholder government agencies at three provinces. We analyzed the power of stakeholders and their attitudes towards reform and briefly discussed how the public hospital-centered service delivery system would perform.

The healthcare system in China serves a fifth of the world’s population. Between 1960 and 2018, life expectancy rose from 43 to 76 years; most of this increase can be attributed to economic growth and improvements in public health [2,6]. However, China’s current healthcare system faces fresh challenges in delivering affordable and high-quality care to meet the growing needs and rising expectations of Chinese people [1]. Unlike the health system in the 1960s and 1970s, the service delivery system after the China Economic Reform highlights the role of acute care and hospitals [7]. Although the system is extremely public-hospital-centered, it is often profit-driven [1]. Public hospitals had 89% of total hospital beds and delivered 92% of outpatient visits [8]. In 2016, 78% of China’s total healthcare expenditure occurred in the hospital sector, as opposed to the 29%-to-42% seen in other countries (Japan 40%, Canada-29%, UK-42%, US-34%) [9]. Because the government set fixed low rates for services and room and board [10], public hospitals were often motivated to maximize profits and use profits to subsidize staff salaries [8].

There were many attempts to improve the public hospital system, but two landmark reforms stand out. A reform in the 1980s introduced market incentives to public hospitals, just like those offered to other sectors in the economy [1]. The number of hospitals in China has more than doubled between 1980 and 2010 [8], and 30% to 40% of hospital revenues were generated by out-of-pocket payments. A more recent reform aims to improve the governance of public hospitals so the public healthcare delivery system can provide affordable, equitable access to healthcare services.

The separation of ownership and operation or *guanban fenkai* is one of the core guidelines for the public hospital reform. In contrast to private hospitals in China or public hospitals in the USA and Europe, the ownership status and legal structure of China’s public hospitals are less clear. Many government branches at different levels are involved in personnel management, financing hospital services, and infrastructure investment decisions [10]. For example, hospital assets are owned by one or more government departments, and hospital administrators have little autonomy in managing their staffing [11]. The lack of separation between ownership and management means that China’s public hospitals are sometimes bureaucratic in culture, structure, and function [2,10].

There are various forms of separation reforms in theory and in practice [2,5,8,12]. The progress of the reform has been slow to date [1,2,8]. To our knowledge, no study has examined why the public hospital reform lacks progress towards improvements in the governance of public hospitals and affordable and high-quality care. This is the first comprehensive study to date examining the reasons behind stymied public hospital governance reform in China. In addition, this study contributes to the literature in two other aspects. First, it gives a voice to public hospital administrators and government stakeholders expressing their concerns and feelings and ensures that study findings of separation reform are grounded in their experiences. Second, it explores the changing power and position of stakeholder groups during the reform.

## Materials and methods

In early 2016, a research center of China National Health Commission was instructed to study the progress and challenges in implementing the public hospital governance reform. The leadership of China National Health Commission wanted to collect opinions and feedback from public hospital administrators and government stakeholders. A total of 32 workshops were carried out at various locations (Table 1). The project team are independent qualitative researchers from the research center and a prestigious university, not affiliated with any stakeholder groups. They have received government funding to organize the workshop meetings. They purposively selected participants from different stakeholder groups and also from various administrative hierarchy levels [10,13]. The research questions in this manuscript were embedded in meetings which lasted for 1.5 to 3 hours. Because invited hospitals and stakeholders received direct orders (meeting announcements) from their upper level government, refusals to participate was not an issue in this study. The ethics committee of Peking University waived the need for ethical approval. The need for consent was waived by the ethics committee of Peking University.

**Table 1:** Workshops and participants.

Workshops #	Geographic representation	Category	Particpants			Month and Year
1	All China	Public hospital administrators	20 from affiliated hospitals of National Health Commission			Feb 2016
2	Beijing		14 from affiliated hospitals of Beijing Municipal Health Commission			May 2016
3	Eastern China		17 from eastern China			Jun 2016
4	Central China		15 from central China			Aug 2016
5	Western China		13 from western China			Sep 2016
			**Provincial level**	**City level**	**County level**	
6–14	a Eastern province	Health Officials	2	2	3	Oct 2016 –Nov 2016
		Public hospital administrators	3: general hospital(1), TCM hospital(1), MCH hospital(1)	6: general hospital(2), TCM hospital(2), MCH hospital(2);	6: general hospital(2), TCM hospital(2), MCH hospital(2);	
		All officials	9: Leadership(1), Org Dept(1), Staffing(1), Pricing(1), HRSS(1), DRC(1), Finance(1), Health(2)	10: Leadership(1), Org Dept(1), Staffing(1), Pricing(1), HRSS(2), DRC(1), Finance(1), Health(2)	14: Leadership(1), Staffing(2), Pricing(2), HRSS(3), DRC(1), Finance(2), Health(3)	
15–23	a Central province	Health Officials	3	3	3	Dec 2016 –Jan 2017
		Public hospital administrators	3: general hospital(1), TCM hospital(1), MCH hospital(1)	6: general hospital(2), TCM hospital(2), MCH hospital(2);	6: general hospital(2), TCM hospital(2), MCH hospital(2);	
		All officials	12: Leadership(2), Org Dept(1), Staffing(1), Pricing(1), HRSS(2), DRC(1), Finance(1), Health(3)	10 prefecture city level government officials from Leadership(1), Org Dept(1), Staffing(1), Pricing(1), HRSS(2), DRC(1), Finance(1), Health(3)	14 county level government officials from Leadership(1), Staffing(2), Pricing(2), HRSS(3), DRC(1), Finance(2), Health(3)	
24–32	a Western province	Healthcare Officials	3	3	3	Mar 2017 –Apr 2017
		Public hospital directors	3: general hospital(1), TCM hospital(1), MCH hospital(1)	6: general hospital(2), TCM (2), MCH hospital(2);	6: general hospital(2), TCM hospital(2), MCH hospital(2)	
		All officials	12: Leadership(2), Org Dept(1), Staffing(1), Pricing(1), HRSS(2), DRC(1), Finance(1), Health(3)	11: Leadership(1), Org Dept(1), Staffing(1), Pricing(1), HRSS(2), DRC(1), Finance(1), Health(3)	13: Leadership(1), Staffing(2), Pricing(2), HRSS(2), DRC(1), Finance(2), Health(3)	

Notes:

Leadership        Offices of Party Secretary and Government Leader

DRC        Development and Reform Commission

HRSS        Human Resource and Social Security (Social Health Insurance)

Org Dep        Organization Department of Chinese Communist Party.

Pricing        Bureau of Price Supervision

TCM        Traditional Chinese Medicine

MCH        Maternal and Child Health.

The participants included 124 hospital administrators (presidents or party secretaries), 25 health officials, and 80 government officials from other departments (Offices of local leadership, Development and Reform Commission, Finance, Human Resource and Social Security, Organization Department of Chinese Communist Party, Bureau of Price Supervision, Office of Staffing). The order, time, and participants of these workshops are listed in Table 1. During the first phase, five workshops were conducted with 79 administrators of general or specialty public hospitals from 30 provinces, autonomous regions, and municipalities. For the second phase, researchers visited three provinces and organized workshops at local governments of three levels (province, city, and county). We organized three workshops at each local government with different stakeholder groups. The workshops with health officials were carried out before the meetings with hospital administrators and other government officials.

We used a descriptive theoretical approach to understand the relationships between the governance reform and its stakeholders [14]. Audio-recording was not used, to facilitate participants involvement in the workshop and stimulate discussions. All participants were stakeholders to the public hospital reform [10], which enhanced the quality and credibility of both the workshop and its results [14]. The facilitator had extensive experience in designing and facilitating workshops with stakeholders in the healthcare system. The facilitator ensured that the group did not deviate from the semi-structured agenda. The shy and quiet participants in the group were protected and encouraged to speak out. Two researchers took notes. Also, notes were confirmed through the feedback process at the end of the workshop. After the workshops, we conducted follow-up one-on-one interviews with "typical participants" and participants with unusual viewpoints or experiences to expand the coverage of the research topic [15]. The process of the workshop can be seen in Table 2.

**Table 2:** Process of the workshop.

Step 1	Step 2	Step 3
Preparation 1. Identify stakeholders based on literature and China’s past experiences. 2. Collaborate with the government to send invitations and discussion questions. Assure potential participants that the results are de-identified.	Workshops 1. Welcome and presentations (10–15 minutes) The research team again affirmed that the study did not record personal information or audio-tape the discussions and encouraged openness in the discussions. 2. General discussions of the questions in the invitation 3. Moderated group discussions 4. Wrap up and break (10–15 minutes) 5. Feedback, reflections, close and thanks	Follow-up discussions with selected participants (~60 minutes)

Hospital administrators were sent five questions in the invitation mail. *1*. *How do you or your staff view the ownership of the public hospital*? *2*. *What are the central objectives of public hospitals from the societal perspective*? *3*. *What government departments are involved in the operation of public hospitals*, *and what do they do*? *4*. *Which of the above management tasks do you think can be transferred to you*? *5*. *If the ownership of your hospital is transferred to a regional new legal entity*, *what do you think will happen*? *If there are problems*, *how to solve them*? Government officials including health officials were also sent five questions in the invitation mail. *1*. *What are the central objectives of public hospitals from the societal perspective*? *2*. *How do you view the ownership of the public hospital*? *How is ownership represented*? *3*. *What functions of your department are related to the central objectives of public hospitals*? *4*. *What specific administrative relationships exist between your department and public hospitals*? *5*. *How can these administrative relationships and tasks be transferred to public hospitals or a new legal entity*? *Would you please discuss the feasibilities of this type of change*? We first asked whether they support creating a new organization that merges ownership-related administrative functions. Then we used those questions in the invitation letter for the general group discussions. For the second half of the discussions, the facilitator used specific probes to fully elicit participants’ views on the power and positions of stakeholders in the governance reform. Those participants spoken at least twice in the discussions were defined as active participants in this study.

Although the most common analyses of workshop results involve a transcript of the discussion, we were not able to transcribe the discussion because audio-recording was not used. Instead, we used a qualitative content analysis to examine the researchers' notes. The analysis focused on extracting themes from the notes, with a deductive approach [16]. One researcher read through the notes several times and created a pilot list of prior themes. The notes were then coded according to these themes. If relevant notes could not be coded into an existing theme, a new theme was created. During the coding process, sub-themes were also created, or existing themes were merged. The coding and analysis results were discussed with a second researcher to improve the extraction of the information and the reliability of the results. Based on the results from 32 workshops, we analyzed the administrative authority, workload, and positions of stakeholders and briefly discussed how the healthcare system would perform.

## Results

A total of 229 hospital administrators and government officials participated in the study. Among them, 33, 31, and 41 officials serve at provincial, city, and county level governments, respectively. The hospital administrators had an average age of 51.0 (95% CI: 43.9–58.1), and over 80% of them (95% CI: 71.8%-88.2%) had post-graduate education. Ten stakeholder groups were considered core influential groups, including local leadership (offices of Party Secretary and Government Leader, e.g. party secretary and governor of a province), Department of Finance, Health Commission, Human Resource and Social Security, Development and Reform Commission, Bureau of Price Supervision, Organization Department of Chinese Communist Party, medical colleges, healthcare professionals, and administrators in public hospitals. They possessed two or three of the following stakeholder attributes: (1) power to influence the reform, (2) the legitimacy of the stakeholder's relationship with the reform, and (3) the urgency of the stakeholder's claim on the reform [13]. About 80% of hospital administrators (99 out of 124) and 78% officials (82 out of 105) actively participated the discussions. Officials with high ranks were more active than lower-ranking ones (84.2% or 64 out of 76 vs 51.7% or 15 out of 29, p<0.01).

The public hospital reform has made little progress to separate ownership and administration. About 66% of hospital administrators, 72% of health officials, and less than 10% of other officials support creating a new organization that merges hospital-ownership-related administrative functions. Table 3 lists the theme categories that emerged from the workshops: society's expectations, tasks or role in the governance reform, potential changes of administrative authority and workload, and stakeholder objectives. In addition, some participants discussed their personal objectives or incentives, which will be described below. We found that all stakeholders perceived that their workload would increase in the short term because of the governance reform of public hospitals. There is quite a distance between society's expectations and stakeholder objectives.

**Table 3:** Stakeholder interests, tasks, and societal expectations.

Stakeholders	Expectations in society	Tasks in the governance reform	Change of Administrative Authority	Change of Workload	Stakeholder Objectives
Local Leadership	Taking the reform of public hospitals as a breakthrough to solve the problems of kanbinnan and kaibingui	1. Develop and communicate the vision for the governance reform and clarify the objectives; 2. Work with the health department to develop a comprehensive plan and send reform tasks to various functional departments; 3. Lead the reform and take full responsibilities for its outcomes	Increased	Increased	Reduce the social costs of reform; reduce the impact on interests groups; ensure political stability; benefit political career development; reduce financial burdens; and develop public healthcare system.
Health	Improve the social welfare, quality of care, and efficiency of public hospitals; improve the accessibility of health services and the responsiveness of health systems, and promote the vigorous development of health services.	1. Develop specific reform plans and implement them; 2. Transfer the personnel management and cadre assessment to public hospitals; 3. Strengthen supervision functions.	Reduced	Increased	Public support and understanding from the society and local leadership that the public hospital reform has many challenges; successfully impletment the reform; retain administrative authority over public hospitals
Finance	Assist in the establishment of an efficient and standardized public hospital compensation mechanism to solve the problem of “kanbingui” to the greatest extent.	1. Develop a new public hospital compensation plan; 2. Provide financial support for new medical institutions; 3. Supervise the financial behavior of new medical institutions	Increased	Increased	Ensure the safety of financial funds, save expenses, achieve budget targets, simplify work and reduce workload, and maintain certain administrative authority over public hospitals
HRSS	Ensure that social security funds and medical insurance funds are safe and sustainable, increase the reimbursement rates and work efficiency, and alleviate the problem of kanbingui	Redefine the social security policies of public hospitals and their employees.	Unchanged	Increased	Control the growing expenses of healthcare, expand health insurance coverage, reduce workload, maintain policy continuity, and retain administrative authority over public hospitals.
DRC	Rationalize the relationship with public hospitals and their organizers/owners, and transfer asset rights	1. Transfer the approval authority for large-scale infrastructure projects to the organizer/owner of public hospitals or the public hospital itself or other government units. 2. Transfer the authority to issue and approve certain special funds.	Reduced	Increased	The workload is not increased by the reform of public hospitals, and retain administrative authority over public hospitals.
Pricing	Stabilize and standardize appropriate medical service prices, alleviate the problem of “kanbingui”; and actively cooperate with the reform of public hospitals.	Re-adjust the price of medical services, or completely transfer pricing to public hospitals	Reduced	Increased	Administrative authority is still maintained over public hospitals without increasing the difficulty of work and the workload.
Org Dep	Assist in promoting the reform of the legal status of public hospitals, personnel management, and multi-site practice; Talent activation in healthcare; supervise the health workforce	1. Clarify the new identity of public hospitals and their owners/organizers; reduce administrative bureaucracy; 2. Transfer the performance appraisal of the senior management team to public hospitals and/or their owners/organizers	Reduced	Increased	Maintain certain authorities of personnel management and performance appraisal over public hospitals.
Medical colleges	Increase the control of affiliated hospitals, enhance the goal of social welfare, and improve quality of healthcare	Tightening the relationship with the affiliated hospital.	Uncertain	Increased	Stengthen affiliated hospitals, and use their revenue to improve medical education and research
Healthcare professionals	Professionalism; provide patient-centered care	1. Understand and support the reform of public hospitals. 2. Improve quality of care and bedside manners, and eliminate selfish distractions.	Uncertain	Increased	Higher income and better career development opportunities, improved administrative support
Hospital administrators	Servant leadership, professionalism, and improve management efficiency.	1. Make relevant adjustments to internal management 2. Reduce the cost of change, implement reform policies, and achieve reform goals	Increased	Increased	More administrative power and independence, solid socioeconomic status, and sense of accomplishment

Notes:

Leadership        Offices of Party Secretary and Government Leader

DRC        Development and Reform Commission

HRSS        Human Resource and Social Security (Social Health Insurance)

Org Dep        Organisation Department of Chinese Communist Party.

Pricing        Bureau of Price Supervision

kanbinnan        healthcare too inaccessible

kaibingui        healthcare too expensive

We found that stakeholders can be regrouped into two large categories by their attitudes towards the public hospital reform. Local leadership, hospital administrators, and health commission said that administrators should have more power over the management of public hospitals. Other government departments and healthcare professionals hesitate on the governance reform.

Although local leadership, the health commission, and hospital administrators have similar attitudes towards the reform, they have quite different objectives and power to influence the reform (Table 3). The local leadership wants to maintain the social welfare nature of public hospitals and alleviate the problem of *kanbinnan* (healthcare is too inaccessible) and *kanbingui* (healthcare is too expensive), without significantly affecting the political stability and interests of stakeholders. The local leadership has the greatest power to influence the reform, but the governance reform of public hospitals is not a policy agenda item of top priority and/or urgency (quote 1).

*Quote 1 (local leadership)*: *“Whether in the government annual report or in the city development plan*, *there is less than half a page on healthcare*, *and it is political rhetoric without details*, *the public hospital governance reform may have to give way to other more critical issues…*”

The health commission supports the governance reform because they felt that they were often wrongly criticized by the media and the general public for all of the problems in healthcare, such as *kanbinnan*, *kanbingui*, violence in hospitals, and homemade cancer drugs. They want to change their image through a reform. They aim to draw a line between the health commission and public hospitals and help society understand the complexity of healthcare and the commission’s difficulties regarding limited resources and political power (quotes 2–5).

*Quote 2 (health commission)*: *When we have a meeting*, *some hospital administrators don’t show up; when the social health insurance have a meeting*, *no administrators dare not to attend…*”

*Quote 3 (health commission)*: *"Hospitals affiliated to medical schools are not under our control* …"

*Quote 4 (health commission)*: *“Some hospital administrators have cured local leaders* … *the administrators’ words are more powerful than ours…*”

*Quote 5 (health commission)*: *“Sometimes we want to reject an expansion project*, *but they (a public hospital) have already got the money*, *and sometimes it has been written in the city’s master plan…*”

The hospital administrators have high expectations for the public hospital reform, as the current system has long suppressed its enthusiasm for innovation and significantly constrained its business intellect. At the same time, they felt the reform lacks operability. They are not fully convinced that the management-related administrative power will be transferred to the administration team (quote 6), as the local leadership and health commission still question the administrator team’s motivation for social welfare. The administrators also felt uncertain about how the reform will affect their cadre ranking in the political system (quote 7).

*Quote 6 (health administrators)*: *"Just add another mother-in-law* … *I need to go to a new department now…*"

*Quote 7 (health administrators)*: *“I should be treated as a bureau-level cadre*, *and I can sit in the first class*, *but there is no policy basis*, *it is very troublesome…*”

Other government departments are concerned about increased workload in the short term and reduced administrative authority over public hospitals in the long term (Table 3). They are reluctant to collaborate with the health commission and public hospitals to make changes and transfer certain authorities to administrators (quotes 8–11).

*Quote 8 (Finance) “If the appropriation is approved (like before)*, *then the health industry gets free lunch*, *and the budgeting and auditing department would definitely disagree…*”

*Quote 9 (HRSS) “It’s impossible to get subsidies and stay uncontrolled*. *What should educational and other public institutions think and do*? …”

*Quote 10 (Pricing)*: *“Controlling CPI is also an important task for the government*. *This is a hard indicator…*"

*Quote 11 (Org Dept): “Is the hospital administrator still a cadre of the Party?* … *How do we manage such institutions? …”*

Healthcare providers have little power to influence the reform. They worry that they may lose their tenure status in the political system after the reform. Moreover, they are uncertain about how the reform will affect their income, career development, and work-life balance.

## Discussions

Hospital administrators, healthcare professionals, and eight government branches were the core influential groups in the public hospital reform. Local government leadership, health commission, and hospital administrators were generally supportive of the governance reform of public hospitals. Other stakeholders hesitate on the reform. The supporters have quite different objectives and power to influence the reform. The public hospital reform is not a top priority to the local leadership although they have the greatest power to influence the reform. The health commission does not have the power and resources to strong-arm other government departments. Although the hospital administrators want more management autonomy from the government, they have little power to influence the reform, and they felt the reform lacks operability. Other government departments were unanimously reluctant to collaborate with the health commission to transfer certain authorities to hospital administrators.

The governance reform of public hospitals has four significant obstacles to overcome. First, the interests of most government stakeholders are not aligned with the public interest, which was observed in other socio-economic reforms in China [17–19]. Second, the uncertainties from the public hospital reform have potential negative impacts on the interests of personnel working for government stakeholders and hospitals. People involved in the reform were concerned about the increased workload in the short term and trade-offs they will face. These two obstacles can be overcome through strong leadership and a dedicated team, similar to what has happened in environmental protection in recent years [20,21]. However, the health commission is not able to push this massive reform effort through because they have limited financial resources and political power. Lastly, and ultimately most importantly, the central government and local leaderships did not make public hospital reform a top policy priority.

The public hospital reform has “*to give way to other more critical issues*.” Although China’s per capita nominal GDP ranked 71st among 184 economies, the Chinese government can accomplish challenging tasks through concentrating on important goals (*jizhong liliang bandashi*). For example, China has used performance evaluation as a hands-off strategy to secure top-down control and meet priority targets [22], such as family planning and economic growth. Since 2009, local governments at or beyond county level have been evaluated in five areas including political/ideology work, leadership capacity, work performance, integrity and anti-corruption, and accomplishing major goals [22]. Work performance is measured by economic development, social development, and many other aspects. Healthcare is lumped into social development. The 2017 Communist Party Congress announced three major goals or “critical battles,” namely financial risk mitigation, poverty reduction, and environmental protection. Therefore, the health commission will need the new fuel in the future to speed up the public hospital reform [23].

This study has several limitations. All the participants were recruited from the bureaucracy hierarchy. They are not volunteers, but they may still have some characteristics in common. We were not able to interview healthcare administrators in the private sector. More themes may be discovered if we can involve the private healthcare companies. We were not able to audio-record the workshops, which is a typical practice in projects initiated by the Chinese government. Using audio-recording is an obstacle for participants to express their opinions openly. Instead, researchers took extensive notes in the workshops.

## Conclusions

Progress in public hospital reform has been slow. The governance reform of public hospitals faces significant obstacles. The interests of most government stakeholders are not aligned with public interests. Most stakeholders involved in the reform are not incentivized to collaborate with each other. The health commission at difference level of governments has insufficient political power and limited financial resources to carry out a massive reform. Most importantly, the public hospital reform is not a top policy priority to the central government or local leaderships. The participants were concerns that the health commission had the political support and resources to speed up the public hospital reform. To fulfill the pledge of affordable, equitable access to quality care, Chinese government needs to overcome these significant obstacles in the public hospital reform and provide the health commission more support and resources.

## Abbreviations

DRC: Development and Reform Commission
HRSS: Human Resource and Social Security (Social Health Insurance)
Org Dep: Organisation Department of Chinese Communist Party.
TCM: Traditional Chinese Medicine
MCH: Maternal and Child Health
GDP: Gross Domestic Products
